# Supplementary material for: Causal interplay between lactose intolerance and gut microbiota: a combined bidirectional Mendelian randomization and in vivo validation study
Source: Front Nutr. 2026 Jun 1;13:1803337. doi: 10.3389/fnut.2026.1803337 (PMC13265576; doi:10.3389/fnut.2026.1803337)
Supplement: Supplementary file 1 [file Data_Sheet_1.zip › supplementary materials/Forward/leave one out cross validation/ebi-a-GCST90027624.finngen_R12_E4_LACTONAS.pdf]

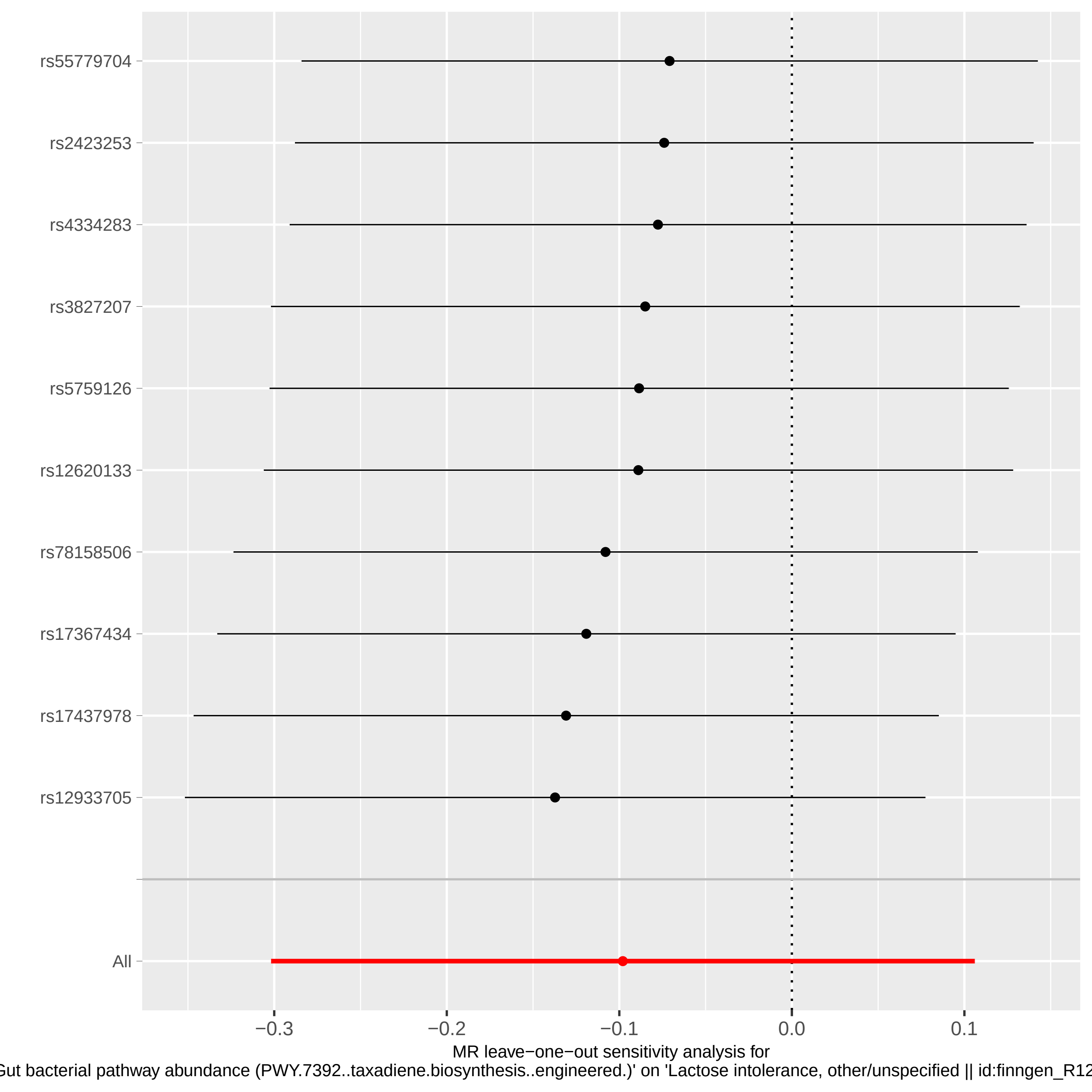
rs55779704
rs2423253
rs4334283
rs3827207
rs5759126
rs12620133
rs78158506
rs17367434
rs17437978
rs12933705
All
−0.3
−0.2
−0.1
0.0
0.1
MR leave−one−out sensitivity analysis for
Gut bacterial pathway abundance (PWY.7392..taxadiene.biosynthesis..engineered.)' on 'Lactose intolerance, other/unspecified || id:finngen_R12
